# The Role of the Receptor Tyrosine Kinase Axl in Carcinogenesis and Development of Therapeutic Resistance: An Overview of Molecular Mechanisms and Future Applications

**DOI:** 10.3390/cancers13071521

**Published:** 2021-03-25

**Authors:** Martha Wium, Aderonke F. Ajayi-Smith, Juliano D. Paccez, Luiz F. Zerbini

**Affiliations:** 1 Cancer Genomics Group, International Centre for Genetic Engineering and Biotechnology, Cape Town 7925, South Africa; mariet.wium@icgeb.org (M.W.); Aderonke.Ajayi@icgeb.org (A.F.A.-S.); 2 Instituto de Ciências Biológicas, Universidade Federal de Goiás, Goiânia 74690-900, Brazil

**Keywords:** Axl, cancer, drug resistance, receptor tyrosine kinase, targeted therapy, molecular mechanisms

## Abstract

**Simple Summary:**

The tyrosine kinase receptor Axl is an oncogene that promotes cancer development by increasing proliferation, survival, invasion, and migration in cancer cells. Axl also contributes to the development of resistance to chemo-, radio-, immune- and targeted therapy in many cancer types. It is a promising therapeutic target, and several inhibitors directed to Axl are currently in clinical trials. The understanding of Axl’s role in the development of resistance can lead to improved and new cancer therapeutic strategies.

**Abstract:**

Resistance to chemotherapeutic agents by cancer cells has remained a major obstacle in the successful treatment of various cancers. Numerous factors such as DNA damage repair, cell death inhibition, epithelial–mesenchymal transition, and evasion of apoptosis have all been implicated in the promotion of chemoresistance. The receptor tyrosine kinase Axl, a member of the TAM family (which includes TYRO3 and MER), plays an important role in the regulation of cellular processes such as proliferation, motility, survival, and immunologic response. The overexpression of Axl is reported in several solid and hematological malignancies, including non-small cell lung, prostate, breast, liver and gastric cancers, and acute myeloid leukaemia. The overexpression of Axl is associated with poor prognosis and the development of resistance to therapy. Reports show that Axl overexpression confers drug resistance in lung cancer and advances the emergence of tolerant cells. Axl is, therefore, an important candidate as a prognostic biomarker and target for anticancer therapies. In this review, we discuss the consequence of Axl upregulation in cancers, provide evidence for its role in cancer progression and the development of drug resistance. We will also discuss the therapeutic potential of Axl in the treatment of cancer.

## 1. Introduction

The development of drug resistance is a major obstacle in cancer treatment. Drug resistance can be defined as a decrease in the efficacy and potency of a medication, implying treatment failure and affecting the patient’s overall survival. The growing prevalence of drug-resistant cancers demands more research into the development of more effective cancer treatments. Some tumours can exhibit intrinsic resistance (i.e., without prior exposure), which can be attributed to several factors such as (a) drug degradation, (b) alteration of the expression or function of the drug target, (c) alteration of the drug transport across the cell membrane, or (d) reduced interaction efficiency between the drug and its target [1,2,3]. In other situations, an initial response to treatment is followed by acquired drug resistance that can be influenced by environmental or genetic factors facilitated by the selection of drug-resistant cell clones or acquisition of mutations in relevant metabolic signalling pathways [1,2,3]. 

The identification of molecular druggable targets is a crucial step for the development of efficient cancer therapies and has a strong scientific and clinical impact. In the last years, investigations have focused on identifying drug targets that are essential for tumour cell viability or immune evasion. In this context, tyrosine kinase receptors were identified as important targets for drug development. 

Tyrosine kinases are significant mediators of the signalling pathways. They play vital roles in biological processes such as proliferation, cell cycle, metabolism, migration, differentiation, and apoptosis [4,5]. Although their activity is strictly regulated in normal cells, they may gain transforming functions due to mutations, overexpression, autocrine and paracrine stimulation resulting in malignancy [4]. This can lead to tumour invasion, metastasis, neovascularization, and resistance to therapy [6]. This review focus on Axl, a receptor tyrosine kinase (RTK) that belongs to the TAM (TYRO3, Axl, and MER) family of RTKs. We discuss its regulation, activation and oncogenic role in cancer development and progression with more attention on its contribution to drug resistance in common solid cancers worldwide, lung, prostate, breast, and liver. Furthermore, we discuss the potential of Axl as an anticancer therapeutic target.

## 2. Axl

Axl was initially discovered during a screen for genes involved in the progression of chronic myeloid leukaemia (CML) to blast crisis [7,8,9]. It is expressed in numerous embryonic tissues and is thought to be involved in mesenchymal and neural development. In adult tissue, its expression is mostly limited to smooth muscle cells [7] and tissue cells that are primed to respond to injuries such as alveolar macrophages, Langerhans cells of the skin and splenic dendritic cells [8]. Axl is involved in various cellular processes including cell growth, proliferation, survival, apoptosis, and adhesion [9]. Given this, the involvement of Axl in cancer progression is not unexpected [9]. It has been associated with different high-grade cancers and correlated with poor prognosis [7] Furthermore, higher levels of Axl expression are found in highly invasive cancer cell lines compared to less invasive cancer cell lines indicating an association with migration and invasiveness of cancer cells. 

**Figure 1:**
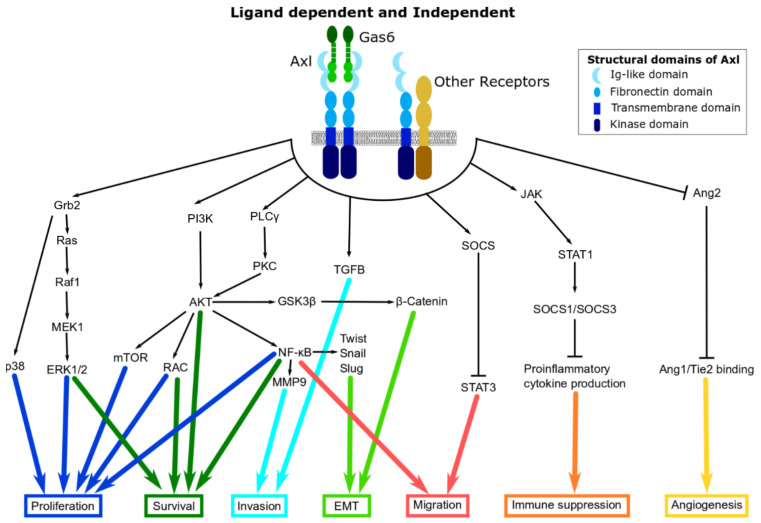
Structure of Axl and its downstream signalling pathways. Various downstream signalling networks can be activated either through the binding of GAS6 (green) to Axl (blue) or through Axl’s interaction with other receptors (yellow).

Structurally, Axl is composed of an extracellular, transmembrane, and intracellular domain [10]. Similar to other TAM family members, the extracellular domain has two immunoglobulin (Ig)-like domains and two fibronectin III domains [9,10,11,12,13] that are involved in ligand binding (Figure 1). The ligand growth arrest-specific protein 6 (GAS6) is the universal ligand for all three TAM receptors, however, it has the highest affinity for Axl [12]. Another universal ligand of the three TAM receptors is Tubby-like protein 1 (Tulp1) [14]. The intracellular domain is the tyrosine kinase domain and has important auto-phosphorylation sites [10]. The tyrosine kinase domain conveys the oncogenic capacity and can be activated with or without extracellular stimulation. 

### 2.1. Regulation of Axl

The synthesis of Axl is controlled by different processes including, epigenetic (via methylation) mechanisms, transcription, translation, and posttranslational modifications. Transcription factors that act on the Axl promoter include YES-associated protein 1 (YAP1), myeloid zinc finger 1 (MZF1), activator protein 1 (AP1), Sp1/Sp3, hypoxia-inducible factor 1 α (HIF1α) and c-Jun [9,10,11,13,15]. Yes-associated protein (YAP) is a downstream effector of the Hippo signalling pathway that regulates organ expansion and tissue development. Axl has been identified as an important downstream target that propels YAP-dependent oncogenic functions [16]. YAP1 and MZF1 transcription factors regulate Axl transcription and increase epithelial-to-mesenchymal-transition (EMT). MZF1 has been shown to bind to Axl promoter leading to the enhancement of Axl transcription in cervical and colorectal cancer [9]. Conversely, MZF1-mediated migration and invasion are reduced by the knockdown of Axl indicating that MZF1 regulate these processes through Axl [17]. The transcription factor AP1 was found to upregulate Axl levels in cell lines resistant to tyrosine kinase inhibitor (TKI) [15] and PI3K inhibitor [18] contributing to the development of resistance. The Sp zinc-finger transcription factors Sp1 and Sp3 can upregulate Axl transcription by binding to the GC-rich Axl promoter region, while Axl gene expression is inhibited by methylation of CpG sites in Sp binding regions [13,15]. Activation of epidermal growth factor receptor (EGFR) pathways and MEK/ERK signalling lead to c-Jun mediated Axl mRNA expression in non-small cell lung cancer (NSCLC) and head and neck squamous cell carcinoma (HNSCC) [19]. Activation of toll-like receptor (TLR) signalling upregulates Axl mRNA in dendritic cells and macrophages. Axl transcription is also regulated by specific mutated forms of p53. For instance, R175H, R273H, and D281G mutated p53 isoforms in H1299 lung cancer cells upregulated Axl mRNA and protein levels [11,20]. 

In addition to transcription factors, Axl is also regulated by the methylation of mRNA. Methyltransferase 3 (METTL3) is a key constituent of the m6A methyltransferase complex, and it has been shown to stimulate Axl translation and EMT, thereby supporting ovarian cancer initiation and progression [21]. 

Axl expression is also controlled by epigenetic modifications [10,11]. Axl promoter region is rich in GC repeats which are necessary for the epigenetic regulation of Axl expression via methylation of cytosine nucleotides [22,23]. 

Posttranscriptional regulation by miR-34a and miR-199a/b have been shown to prevent translation of Axl mRNA in several cancers by binding to its 3′-UTR [13]. A recent study done in our laboratory demonstrated that miR-7 and miR-34a act as regulators of Axl in prostate cancer. These miRNAs expression levels are inversely associated with Axl expression in clinical prostate cancer samples [24]. Furthermore, we demonstrated a novel regulation pathway for Axl, mediated at least partly by inhibition of miR-34a and miR-7. Axl expression relies on JARID 2 and EZH2, components of the Polycomb Complex Repressor 2 (PRC2). The PRC2 is a complex of proteins involved in proliferation, pluripotency, and maintenance of the developmental stage in adults. This complex regulates the chromatin structure mainly by methylation of histone H3 lysine 27 residue (H3K27) [24].

The long non-coding RNA (lncRNA), differentiation antagonizing non-protein coding RNA (DANCR) was shown to upregulate Axl through competitive binding to miR-33a-5p, which targets Axl mRNA for degradation [25]. Cancer metastasis-associated long intergenic non-coding RNA (CALIC) has been shown to associate with the RNA-binding protein heterogeneous nuclear ribonucleoprotein-L (hnRNP-L) to upregulate Axl, leading to the promotion of migration and metastasis in colon cancer cells [26]. 

Axl protein is approximately 120 kDa to 140 kDa in size depending on the level of posttranslational modifications by glycosylation, phosphorylation, and ubiquitination [15,27]. Phosphorylation activated Axl while ubiquitination marked proteins degradation by the lysosome.

### 2.2. Axl Activation

Axl is believed to perform its role in adhesion through its fibronectin domains [9,15]. These domains are similar to that of adhesion molecules such as neural cell adhesion molecule (NCAM) and L1 [15,28]. The binding of GAS6 to Axl was shown to have a positive impact on cell-cell adhesion [15]. The activation of Axl and downstream signalling pathway depends on various mechanisms. The typical method of Axl activation in physiological conditions is ligand-dependent homodimerisation; however, Axl can also be activated by several ligand-independent mechanisms [22]. Ligand-dependent leads to the activation of Axl via transautophosphorylation of numerous tyrosine residues in the intracellular domain of the protein. The activation of Axl is only complete when it interacts with the phospholipid phosphatidylserine, which is facilitated by the gamma-carboxyglutamic acid (Gla) domain on GAS6 after its posttranslational modification [9]. 

Ligand-independent activation of Axl has also been described. For instance, in MCF-7 breast cancer cells, activation of Axl stimulated NF-κB mediated activation of matrix metalloproteinase-9 (MMP9). However, this activation is possible with an Axl mutant that cannot bind GAS6 and is not enhanced by GAS6 overexpression, indicating that Axl can be activated independently from its ligand [29]. Overexpression of Axl can lead to self-dimerization (homodimerization) and activation in NIH 3T3 mouse embryonic fibroblast cells [30]. Axl can also be activated by heterodimerization with EGFR and other tyrosine kinase family members including hepatocyte growth factor receptor (c-Met), platelet-derived growth factor receptor (PDGFR), human epidermal growth factor receptor 2 (HER2) and HER3 [31,32]. Axl-EGFR heterodimers promote invasion by upregulation of MMP9, diversifying signalling pathways beyond those triggered by either Axl or EGFR homodimers alone [32]. Axl can also be activated through the extracellular domain-mediated dimerization with HER2 promoting cell invasion and metastasis in mice [33]. 

Oxidative stress can activate Axl phosphorylation. The activation of Axl by H_2_O_2_, a reactive oxygen species (ROS) may be mediated partly via an increased intrinsic tyrosine kinase activity of Axl or decreased tyrosine phosphatase activity [34]. In mesothelioma cells, ROS induced Axl phosphorylation which was consequently inhibited by BGB324 a selective inhibitor of Axl [35].

## 3. The Role of Axl in the Hallmarks of Cancer 

The hallmarks of cancer encompass biological abilities acquired during the multistep development of human cancer. They include evading growth suppressors, sustaining proliferative signalling, inducing angiogenesis, resisting cell death, enabling replicative immortality, activating invasion and metastasis, evading immune destruction, and reprogramming of energy metabolism [36]. Axl controls processes necessary for both cancer development and aggressiveness in many human malignancies [37]. Axl signalling has been shown to promote several cancer hallmarks including proliferation, survival, invasion, migration, inhibition of apoptosis and cellular adhesion in cancer cells [12]. 

### 3.1. Proliferation and Survival 

Axl promotes cell proliferation through signalling pathways such as the PI3K/AKT/mTOR, RAS/RAF/MEK/ERK (also known as the MAPK pathway), JAK/STAT and NF-κB (Figure 1). It promotes cell survival by regulating the nuclear translocation of NF-κB resulting in the increased expression of anti-apoptotic markers such as survivin and B-cell lymphoma 2 (BCL-2), and a concurrent decrease in the activity of pro-apoptotic proteins such as caspase 3 and BCL-2 antagonist of cell death (BAD) [10]. The association of Axl with the MAPK pathway has been described in some studies. For instance, ligand-dependent Axl activation has been shown to stimulate MAPK as well as AKT and FAK pathways in NSCLC cell lines [38]. In DU145 prostate cancer cell line, the P13K/AKT and MAPK pathways were shown to be involved in Axl/GAS6-induced proliferation [39]. In prostate cancer, the mechanistic consequence of Axl inhibition results in the inactivation of the NF-κB pathway through inhibition of AKT. The inhibition of NF-κB kinase subunit alpha (IKKα) activity blocks the interleukin 6 (IL-6)/STAT-3 signalling pathway leading to a decrease in proliferation, migration, and invasion by inducing apoptosis of prostate cancer cells and inhibits tumour formation in a xenograft mouse model [40]. Similarly, the inhibition of Axl resulted in the inhibition of AKT and MAPK pathways suggesting that these pathways are involved in Axl-induced growth and proliferation in acute myeloid leukaemia cells [15]. The suppression of Axl in chronic lymphocytic leukaemia (CLL) reduced levels of the anti-apoptotic protein MCL-1 and stimulate apoptosis [41].

### 3.2. Invasion and Migration 

Axl knockdown by RNA interference (RNAi) causes a decrease in the migration and invasion in various cancer types including liposarcoma, lung adenocarcinoma, breast, pancreatic and thyroid cancer, providing evidence for the role of Axl in conferring migratory and invasive characteristics [10]. The overexpression of Axl in cancer cells with low metastatic potential promotes migration and invasion [12] and it is a driving force in the spread of tumours in vivo and in vitro [10,42]. The activity of Axl is necessary for phenotypes associated with cell migration including the increase in the GTP-binding protein Rho and Rac and the formation of filopodia [10]. Axl activation stimulates the expression of p-AKT and MMP9 (an essential effector of invasion) through the activation of NF-κB and Brg-1 [10,12]. Activation of Axl increased invasion and migration in Oral squamous cell carcinoma (OSCC) cell lines [37] and activated AKT in advanced ovarian tumours. It triggers the PI3K/AKT pathway and activates the expression of proteolytic enzymes such as MMP2 and MMP9 destroying the extracellular matrix and increased cancer cell invasion [12]. In Glioblastoma cells expressing high basal levels of Axl induced by GAS6, the knockdown of Axl led to reduced response to sunitinib (multi-targeted TKI) by rescuing migration [43]. In mesenchymal triple-negative breast cancer cells, Axl is localized to the Golgi apparatus and the leading edge of the migrating cell [44]. This polarization at the leading edge can be displaced by the Axl inhibitor, R438 and may indicate that Axl controls directed cell migration [44]. The knockdown of YAP1 significantly reduced invasion in lung adenocarcinoma through the downregulation of the Axl pathway [11]. In YAP1-transformed MIHA HCC cells, the knockdown of Axl interfered with migration and invasion and reduced the metastatic potential of the cells [16]. Another study done using melanoma cancer cell lines showed that Axl is an important gene in YAP-induced melanoma cell invasion [45]. 

In addition, Axl is associated with the expression of stem cell markers and regulates metastases genes (Sstr2, Flt4, MMP10, Kiss1, MET, Col4a2, RORB) and plays a role in breast cancer stem cells migration and invasion [46]. In oesophageal adenocarcinoma cell lines, Axl plays a role in the peripheral distribution of lysosomes and in the production of cathepsin B, which promotes invasion [10]. 

### 3.3. EMT 

Numerous studies describe the pro-carcinogenic role of Axl in promoting EMT, Figure 1 [9,10,46,47]. Activation of Axl enables cells to maintain a mesenchymal phenotype that increases cell invasiveness and drives metastasis [7,10]. In breast cancer stem cells, increase Axl levels induces EMT by controlling the expression of EMT markers such as E-cadherin and N-cadherin, and increasing EMT-associated transcription factors such Snail, Slug, and Twist [46]. This is supported by the fact that stable knockdown of Axl leads to the downregulation of EMT-associated transcription factors, Slug, Snail, and Twist and reduced migration and invasion in pancreatic and OSCC models [8,48]. Interestingly, transfection of Slug and Snail into MCF10A cells (human breast epithelial cells) is linked to increased expression of Axl as well as lead to increasing mesenchymal-type markers and decreasing epithelial-type morphological characteristics [10], establishing a positive feedback loop between Axl and Slug/Snail [49]. 

### 3.4. Angiogenesis 

Angiogenesis is a normal physiological process during wound healing, tissue reconstruction and repair but it promotes tumour growth, expansion, and metastasis by providing oxygen, nutrients, and hormones to cancer cells [10]. Axl overexpression in neoplastic setting increases angiogenesis; the tumour microenvironment is rich in reactive oxygen species (ROS) may contribute to this by increasing Axl activation [22]. Axl knockdown on the other hand results in the downregulation of Dickkopf-related protein 3 (DKK3) and angiopoietin-2 (Ang-2). DKK3, a member of the Dickkopf family involved in Wnt signalling [22], controls endothelial tube formation. Overexpression of DKK3 in the C57/BL6 melanoma model leads to increased microvessel density. Ang-2 prevents the association of Ang-1 with Tie2 that together drive endothelial cell survival. The downregulation of Ang-2 through Axl activation frees Ang-1 and Tie2 thus allowing their proangiogenic activity [22,50]. 

### 3.5. Stem Cell Maintenance 

Cancer stem cell phenotype contributes to resistance to anti-cancer treatment, promotes metastasis and tumour latency. Axl has been implicated as a key factor in promoting cancer stem cell phenotype [51]. Axl expression correlates with the expression of cancer stem cell markers such as Bglap1, Cdc2a, CD44, and ALDH1. CD44 and ALDH1 increase have been shown to increase the resistance of cutaneous squamous cell carcinoma to chemotherapy [10,51]. Axl expression in human glioblastoma is associated with high EZH2 expression which plays an important role in stem cell maintenance [10]. Axl is important in the invasion and migration of breast cancer stem cells (BCSCs) [46]. Treatment with the Axl inhibitor MP470 (Amuvatinib) reduced the mammosphere forming ability of BCSCs and increased response to chemotherapy [46,51]. Axl is selectively overexpressed in CML CD34^+^ cells, and its knockdown resulted in decreased survival and self-renewal ability of human CML CD34^+^ cells. Furthermore, the suppression of Axl by shRNA knockout and therapeutic inhibition increased the survival of CML mice and decreased the growth of leukaemia stem cells in mice [52].

### 3.6. Immune Checkpoint

In acute myeloid leukaemia, Axl activation is involved in immune evasion via the upregulation of BCL-2 and Twist, the suppression of TLR inflammatory signalling and the limited expression of pro-inflammatory cytokines [53,54]. Axl plays a role in radio-resistant and checkpoint immune-resistant tumours through the suppression of antigen presentation through MHC-I and enhancement of myeloid-supporting cytokines and chemokines leading to an inadequate initial immune response [10,54]. Axl has been shown to upregulate the expression of the immune checkpoint molecule, programmed death-ligand 1 (PD-L1) in head and neck cancers [55]. In metastatic melanoma patients, genomic and transcriptomic data suggest that Axl overexpression may play a role in innate sensitivity or cause resistance to anti-programmed cell death protein 1 (PD1) therapy [10]. In the mouse xenograft model of the radioresistant MMTV-pyMT breast cancer, the genetic knockout of Axl resulted in reduced growth and increased sensitivity to radiation therapy and immunotherapy, which was associated with about twenty-fold increased CD8^+^ T-cell response [56]. These results suggest a role for Axl in suppressing antigen presentation via MHC-1 and altering cytokine release leading to insufficient immune response and T-cell exclusion [56]. Altered cytokines include macrophage recruiting chemokines, CCL3, CCL4, and CCL5 and NF-κB target cytokines, IL-6, TNF-α, and IL-1 α [56].

## 4. Axl in Drug Resistance

In the last decade, several reports demonstrated a clear role of Axl in the development of resistance to anticancer therapies. Axl is involved in the resistance of drugs with different mechanisms of action, ranging from cytotoxic drugs to tyrosine kinase inhibitors, Table 1. In this section, we will discuss the role of Axl in the resistance to different drugs in the context of five of the most common types of cancer, lung, breast, prostate, and liver.

### 4.1. Lung Cancer

Lung cancer is the most diagnosed and lethal cancer representing 11.6% and 18.4% of all cancers accordingly to GLOBOCAN 2018 [46,78,79]. The most prevalent forms of lung cancer are non-small-cell lung carcinoma (NSCLC) and small-cell lung carcinoma (SCLC) that represent 80% and 18% of all types of lung cancer, respectively [80]. Despite multimodality treatment strategies which include surgery, radiotherapy, chemotherapy, and targeted therapy, the 5-year survival rate is only 20% [79,81]. This is mostly because of late diagnosis, with only 20% of NSCLC cases resectable at diagnosis, and the development of drug resistance. 

Frequent mutations in the EGFR have led to the development of TKIs that include dacomitinib, erlotinib, afatinib, osimertinib, and gefitinib. These drugs have been employed in clinical use as first/second treatment line for EGFR-mutated lung adenocarcinoma patients leading to response rates and progression-free survival (PFS) ranging from 56% to 83% and 8.4‒18.9 months [82,83,84,85].

**Figure 2:**
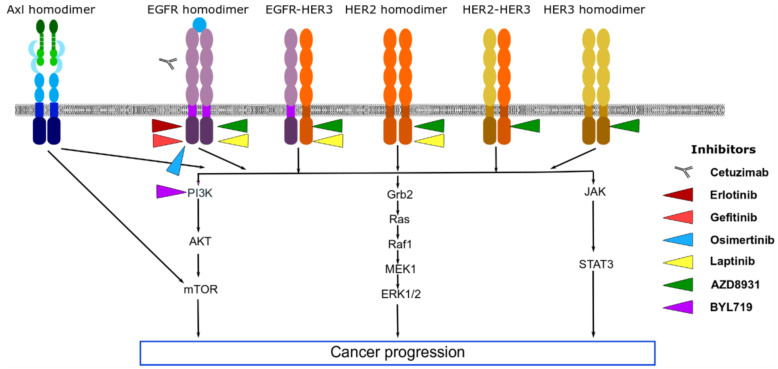
The role of Axl in resistance to epidermal growth factor receptor (EGFR), human epidermal growth factor receptor 2 (HER2) and HER3 tyrosine kinase inhibitors.

Axl has been described to be an important player in the mediation of TKI-resistance, Figure 2. Zhang et al. [62], using both in vivo and in vitro models, demonstrated the activation of Axl in EGFR-mutant lung cancer models cause resistance to erlotinib [62]. In line with these observations, Byers et al. [86] validated a 76-gene EMT signature using gene expression profiles from NSCLC cell lines and patients treated with EGFR inhibitors [86]. They identified Axl as a central mediator of resistance to EGFR inhibitor associated with mesenchymal phenotype and that the inhibition of Axl in mesenchymal cells reverses the acquired resistance to the drug treatments [86]. 

Recently, Wang et al. [87], evaluated the effect of Axl inhibitors; glesatinib, sitravatinib, and BGB-324 in monotherapy or in combination with erlotinib on cell cycle progression and apoptosis in EGFR TKI-resistant NSCLC cells [87]. They observed that the combination of Axl inhibitor with erlotinib reduced cell growth while inducing G2-M cell cycle arrest and enhancing apoptosis when compared to single-agent treatment [87].

In the EGFR TKI-resistance patients Axl may activate downstream pathways inducing cell survival and growth, circumventing the need for EGFR activation, and suggesting that co-treatment of EGFR and Axl inhibitors may reduce tumour growth more effectively [88]. Gefitinib, an EGFR inhibitor, is used for the treatment of NSCLC in patients with EGFR-activating mutation. Analysis of the Axl status in tumour tissue samples of patients that showed an initial response to gefitinib prior to resistance showed a 20% increase in Axl level following gefitinib treatment [88]. In another study using a pair of sensitive and gefitinib-resistant cell lines, Tian et al. [89] had demonstrated that gefitinib-resistant cells overexpress Axl and its downstream targets [89]. Silencing of Axl in the gefitinib-resistant cells restored the effect of the compound and Axl overexpression conveys resistance to gefitinib in sensitive cells [89]. Recently, Du et al. [90] identified miRNA-625-3p as an important player in gefitinib resistance. Using NSCLC-derived cells and the corresponding gefitinib-resistant cell lines, they observed decreased expression of miRNA-625-3p in gefitinib-resistant cell lines and identified Axl as a miRNA-625-3p target [90]. Both Axl knockdown or miR-625-3p overexpression could reverse the gefitinib resistance phenotype [90].

Interestingly, yuanhuadine (YD), a natural product-derived antitumour agent, inhibits Axl and overcame gefitinib resistance in NSCLC cell model [91]. The combination of gefitinib and YD treatment in gefitinib-resistant NSCLC cell lines demonstrate synergistic growth-inhibition by downregulating Axl expression [91]. 

Osimertinib (EGFR TKI) is used to treat patients with EGFR-mutated lung cancer and as observed for other TKIs, some patients demonstrated intrinsic resistance with insufficient response to the drug [92]. In a recent study, Taniguchi et al. [58] provide new insights into the role of Axl in intrinsic osimertinib resistance. Their findings demonstrated that osimertinib stimulated Axl by inhibiting a negative feedback loop that involved the suppression of SPRY4, a known tumour suppressor in lung cancer, that acts as potent RTK inhibitors. The treatment of PC-9 NSCLC cells showed an increase in the phosphorylation of HER3, c-Met and Axl. In the presence of osimertinib, the knockdown of c-Met did not affect cell viability, conversely, the knockdown of HER3 and Axl decreased the viability of PC-9 cells [58]. Knockdown of both HER3 and Axl decreased cell viability as effectively as the knockdown of *EGFR* and *Axl*, pointing to an interaction between Axl and EGFR or HER3 thereby indicating that the activation of Axl may occur by heterodimerization with other RTKs [58].

Interestingly, Axl is more frequently overexpressed in lung adenocarcinomas that display EGFR-activating mutations, when compared to those that have wild-type EGFR [93]. In H358ER erlotinib-resistant human lung adenocarcinoma cell line, with wild-type EGFR the overexpression of Axl was reported, however, the knockdown of Axl in this cell line did not restore sensitivity to erlotinib [94]. This observation suggests that the role of Axl in resistance to EGFR TKI might only be significant in instances where EGFR is mutated.

Axl is also involved in the development of resistance to other molecules such as monoclonal antibodies. The anti-EGFR monoclonal antibody, cetuximab, is effective in NSCLC treatment [95,96], however, resistance to this therapy has been described in patients [97]. Interestingly, Axl was observed to be upregulated in cetuximab-resistant cells and xenograft tumour model and inhibition of Axl restored the sensitivity to cetuximab [97].

Terry et al. [98] demonstrated that Axl is upregulated in mesenchymal lung cancer clones and its expression correlated with resistance to natural killer (NK)- and cytotoxic T lymphocyte (CTL)-mediated killing [98]. These authors demonstrated that pharmacological targeting of Axl re-sensitized mesenchymal lung cancer cells to CTL-mediated killing. Attenuation of Axl-dependent immune resistance involved a complex network involving NF-κB activation, ICAM1 expression, and inhibition of MAPK [98].

### 4.2. Prostate Cancer

Prostate cancer is the most common type of non-cutaneous cancer and is the second most frequent cause of cancer mortality, being surpassed only by lung cancer [99]. It is the second most frequently diagnosed cancer, in addition to representing the fourth most lethal cancer in men [79]. During the development of prostate cancer, cell survival depends primarily on the androgen receptor, as the decrease in the levels of these hormones is associated with a gradual transition from prostate cancer in a dependent to an androgen-independent manner, which it is more aggressive and difficult to treat [100]. Surgery, radiation therapy and hormone therapy alone or in combination are the most common therapeutic approaches when the disease is in its early stages. Although these therapies are effective for local hormone-dependent prostate cancer (PCa), they are ineffective in patients with metastatic, castration-resistant PCa (mCRPC). In these situations, the most suitable treatment is the use of docetaxel, a second-generation taxane, derived from *Taxus baccata* [101]. 

Docetaxel-treated patients have an average survival of two years, however, nearly all patients become refractory due to the development of resistance [102]. To elucidate the mechanisms that lead to docetaxel resistance in prostate cancer, Lin et al. [63], established two prostate cancer cell lines resistant to docetaxel using the castration-resistant cell lines DU145 and PC-3. Their findings demonstrated that Axl expression and activation were increased in the docetaxel resistant cells. Also, they showed that the inhibition of Axl using siRNA led to an increase in the levels of apoptosis which reduces the migration and invasion of docetaxel-resistant prostate cancer cells [63]. Interestingly, the antitumour-effect of docetaxel was restored both in vivo and in vitro when Axl expression was suppressed genetically or chemically in docetaxel-resistant cells, pointing to a specific role of Axl in docetaxel resistant phenotype [63]. As far as we are aware this is the most relevant report linking Axl expression/activation and docetaxel-resistance in prostate cancer. Additionally, in a recent publication, we demonstrated that co-treatment with Dihydroartemisinin (DHA), a derivative of Artemisinin commonly used in the treatment of Malaria, synergizes with docetaxel effects in both in vitro and in a xenograft mouse model, indicating that Axl inhibition by DHA may prevent or at least delay resistance to docetaxel in a prostate cancer model.

### 4.3. Breast Cancer

Breast cancer is the second most diagnosed cancer and the leading cause of cancer deaths in women [79,103]. Based on the presence or absence of the Hormone receptors (HRs), progesterone receptor (PR) and oestrogen receptor (ER) and human epidermal growth factor receptor 2 (HER2) breast cancer can be divided into three subtypes: HR^+^ (ER^+^/HER2^−^, or PR^+^/HER2^−^ or ER^+^/PR^+^/HER2^−^), HER2^+^ (HR^+^ or HR^−^), and triple-negative breast cancer (TNBC, ER^−^/PR^−^/HER2^−^). Axl expression has been observed in all the major transcriptional subtypes of breast cancer, and its expression in primary breast tumour is strongly indicative of poor outcome and reduced patient survival [9].

As in lung cancer, Axl plays a role in resistance to EGFR-targeted therapy (examples erlotinib and lapatinib) in TNBC and HER2+ breast [31,64], Figure 2. In TNBC, Axl acts to diversify EGFR-induced signalling by triggering additional pathways that EGFR by itself cannot induce, resulting in resistance to EGFR-targeted therapy [31]. This diversification of signalling pathways is ligand-independent and is triggered by the association of Axl with EGFR, c-Met, and PDGFR [31]. The TKI DCC-2036 suppresses Axl/c-Met-PI3K/AKT-NF-κB signalling to decrease growth and metastasis in a TNBC xenografted model overexpressing Axl [104]. 

In HER2^+^ BT474 breast cancer cell lines, the overexpression of Axl is directly involved in the development of acquired resistance to HER2 inhibitor, lapatinib [64]. The multikinase inhibitor, foretinib as well as RNA silencing of Axl could restore lapatinib and trastuzumab (monoclonal antibody against HER2) sensitivity in the resistance cell lines [64].

An alternative to overcome resistance to EGFR inhibitors associated with high Axl levels is to increase Axl degradation. Heat shock protein 90 (HSP90) acts as a molecular chaperon that stables the Axl protein [78]. The HSP90 inhibitor, 17-allylamino-17-demethoxygeldanamycin (17-AAG) can cause a significant decrease in Axl protein levels in cell line and xenograft models, inhibiting EMT and tumour growth [105]. 

MEK inhibition led to decrease shedding (cleavage) of multiple kinase receptors including Axl in TNBC [106]. The proteins A Disintegrin and Metalloproteinases (ADAM)10 and ADAM17 are responsible for the proteolytic cleavage of the extracellular surface domain of Axl. Reduced cleavage causes increased accumulation of Axl and enhanced Axl signalling that is associated with resistances lapatinib [106].

In chemoresistant breast cancer cells, Axl silencing suppresses invasion and migration, and increase the susceptibility to the chemotherapeutic drug, doxorubicin [107]. The AKT/GSK-3β/β-catenin cascade was responsible for Axl-induced cell invasion by upregulation of ZEB1 in a ligand-dependent manner [107]. ZEB1 regulates DNA repair and doxorubicin resistance. 

Depleting Axl can also restore sensitivity to non-targeted chemotherapy such as etoposide and paclitaxel [46]. The results collectively show that targeting Axl in therapeutic approaches will lead to improved outcomes and reduce breast cancer metastases and recurrence. Aside from its role in the development of resistance to chemotherapy and targeted therapy, Axl has also been implicated in resistance to immunotherapy and radiation therapy [10]. In mouse mammary tumours that showed no response to ionizing radiation used in combination with immune checkpoint therapy, high expression of Axl were found. Likewise, the deletion of Axl from these resistant tumours caused them to become radiosensitive [9]. In xenograft breast cancer models, the genetic knockout of Axl resulted in reduced growth and increased sensitivity to radiation therapy and immunotherapy [56]. Axl contributing to radiation-resistance is also reported in HNSCC where significant overexpression and hyperactivation of Axl was observed in radiation-resistant HNSCC cell-line xenografts and patient-derived xenografts. The inhibition of Axl increased the sensitivity of HNSCC cells to radiation implying that targeting Axl has radiosensitising effects in HNSCC [72].

### 4.4. Liver Cancer

Worldwide, liver cancer is the sixth most common type of cancer) and the fifth most lethal [46,78,79]. Liver cancer is divided into two main different types: Hepatocellular carcinoma (HCC), accounting for 75 to 90% of primary liver cancer, and cholangiocarcinoma [108]. 

While surgery, liver transplant, ablation, chemo and radioembolization and radiation therapy may be effective in the early stages of the disease, the treatment of advanced HCC is challenging. Treatment with cytotoxic agents (5-fluorouracil, cisplatin, doxorubicin, gemcitabine, capecitabine, epirubicin) or combined regimens do not show improved response rates or improvements in overall survival. The TKI sorafenib—the first drug approved for first-line systemic treatment of patients with advanced-stage HCC—has shown an improvement in median overall survival of only 2-to-3 months. Recently, lenvatinib, another multi-TKI, has been approved as a first-line treatment for advanced HCC patients and has demonstrated similar efficacy to sorafenib [109].

In HCC, Axl has been shown to mediate Yes-associated protein (YAP)-dependent oncogenic functions that potentiate migration [16]. Axl is a regulator of the Hippo pathway that stimulate tumour cell invasion [16]. Interestingly and as stated for other types of cancers, in HCC, Axl plays a role in EMT by mediating E-cadherin repression and Vimentin upregulation through control of Slug, Snail and Twist transcription factors [110,111]. In addition, Axl has been shown to regulate TGF-β in HCC. TGF- β plays a dual role of tumour-suppressor and tumour-promoter in the early and advanced stages of HCC [112]. Reichl et al. [110] demonstrated that in advanced HCC with mesenchymal characteristics, Axl regulates the TGF- β and promote tumour migration. Interestingly, Axl knockdown impaired resistance to TGF-*β*-mediated growth inhibition reverting the function of TGF-β to tumour suppressor [110]. 

Li et al. [113], demonstrated that an Axl-specific miRNA enhances sensitivity to the chemotherapeutic agent cisplatin. Using functional tests, they demonstrated that miRNA-34a, a miRNA known to inhibit Axl, decreased proliferation levels and induced apoptosis while decreasing chemoresistance to cisplatin in HCC cell lines [113]. Additionally, Leung et al. [114] demonstrated that the adaptive resistance acquired after sorafenib treatment in HCC cells activates several RTKs including Axl [114]. Furthermore, in a recent report, Pinato et al. [69] demonstrated that Axl activation plays a role in the development of sorafenib-resistance. Using two pairs of sorafenib-naive and resistant clones from two different origins (epithelial and mesenchymal), they demonstrated upregulation of Axl in Sorafenib-resistant cells. Axl inhibition by shRNA led to increased sensitivity to sorafenib in both naïve and resistant cells [69]. Their findings point to an important role of Axl dependent signalling in the resistant phenotype of HCC cells. 

## 5. Axl as a Therapeutic Target 

Axl is a promising therapeutic target that can improve patient outcomes, reduce cancer metastases and recurrence, and reverse resistance. This has led to the development of several small molecular Axl inhibitors, monoclonal antibodies, and CAR T-based (chimeric antigen receptor-modulated T lymphocyte) therapies that are currently investigated both in preclinical and clinical studies, Table 2 and Table 3. Small molecular inhibitors specific to Axl include BGB324/Bemcentinib/R428, SLC-391/SLC-0211, and TP-0903 while INCB081776 and ONO-7475 can inhibit both Axl and MER. These drugs are in different clinical trials phases and some show possibilities of improving patients outcomes [9,115]. 

Although Axl-targeted small molecule tyrosine kinase inhibitors have displayed therapeutic values in some cancers, some of these small molecules show off-target toxicities. Thus, there is a need to identify molecules that can effectively disrupt the Gas6/Axl axis [116]. Antibody therapy specifically targeting Axl has been successful in inhibiting cancer growth in vitro and has progressed to phase I and II clinical [117,118]. These include the antibody-drug conjugates, BA3011/CAB-Axl-ADC and HuMax-Axl-ADC and the Anti-Axl Fc Fusion Protein, AVB-S6-500. A recent study by Duan et al. [119] showed that DAXL-88, a phage-derived monoclonal antibody targeted both human and mice Axl with high affinity and specificity. DAXL-88 inhibited the interaction between Axl and GAS6 thereby reducing migration and invasion in ovarian and lung cancer cells [119]. Similarly, the anti-Axl monoclonal antibody 20G7-D9 has been shown to inhibit signalling, EMT, decrease migration and invasion and tumour growth in TNBC breast cancer xenografts [9].

Nucleic acid aptamers are new treatment molecules made up of short single-stranded RNA or DNA that have high binding affinities to their target molecules. Aptamers have numerous benefits over other treatment molecules because of their reduced toxicity, ease of synthesis, fast tissue penetration, long-term stability, and reduced cost [120]. GL21.T, a 2′fluoro pyrimidine RNA aptamer was shown to bind and inhibit Axl thus preventing migration and invasion, interfering with spheroid formation and inhibiting tumour growth in human NSCLC mouse xenograft model [121]. Recently, a novel DNA aptamer that binds selectively to Axl was shown to inhibit 30–40% cell growth and viability in human lung cancer cells with acquired resistance to EGFR-TKI [120].

## 6. Conclusions and Future Perspectives

Although the development of novel drugs and strategies to battle cancer is in constant expansion, the increase in drug resistance continues to be the principal restrictive factor to cure patients with cancer. As discussed above, Axl is a key player in drug resistance in different cancer types. 

A key area that requires more research would be the stratification of patients for effective Axl inhibitor treatment. It would be pivotal to identify patients that are likely to develop Axl-dependent resistance before starting with treatment, as these patients may benefit most from Axl inhibition. Markers currently under investigation include Axl expression levels, phosphorylation or activation status and presence of ligand, Gas6; however, the approach to patient stratification needs further refinement.

Another area that requires more research is the relevance of Axl in drug resistance and the use of Axl inhibitors in single or combination therapies. Inhibition of Axl may revert resistance to a specific drug which in turn can lead to a reduction in the required dose and off-target side effects. 

To conclude, targeting Axl may present strategies to prevent, overturn or delay the development of resistance, making it a promising therapeutic target. In vivo and in vitro studies investigating Axl inhibitors is ongoing and may lead to the discovery of new therapeutic approaches with improved patient life quality and expectancy. 

## Figures and Tables

**Table 1:** Axl in resistance in cancer treatment.

Cancer Type	Drug (s)	Drug Target (s)	Model (Human, Animal, Cell Line)	Refs.
NSCLC	Erlotinib, Gefitinib	EGFR	Human	[57]
	Cetuximab	EGFR	Cell line	[19]
	Osimertinib	EGFR	Cell line	[58]
	Crizotinib	ALK, c-Met	Cell line	[59]
	Cisplatin	Interferes with DNA damage repair mechanism	Cell line	[60]
	Doxorubicin	Inhibition of DNA topoisomerase II activity	Cell line	[60]
	Etoposide	Inhibition of DNA topoisomerase II activity	Cell line	[60]
	Paclitaxel	Microtubule polymer stabilizer	Cell line	[61]
	Vincristine	Binds to tubulin and inhibits the formation of microtubules	Cell line	[61]
EGFR-mutant NSCLC	Erlotinib	EGFR	Mouse, cell line	[62]
Prostate	Docetaxel	Inhibitor of depolymerisation of microtubules	Cell line	[63]
Breast	Lapatinib	EGFR, HER2	Cell line	[64]
	Erlotinib	EGFR	Cell line	[31]
	AZD8931 (Sapitinib)	EGFR, HER2, HER3	Cell line	[65]
	Fluorouracil	Inhibits DNA/RNA replication	Cell line	[66]
	Paclitaxel	Tubulin Inhibitor (Microtubule polymer stabilizer)	Cell line	[67]
Liver	Erlotinib, Gefitinib	EGFR	Cell line	[68]
	Sorafenib	Raf-1, B-Raf, VEGFR, PDGFR, Flt-3 and c-KIT	Cell line	[69]
Head and neck squamous cell carcinoma	Cetuximab	EGFR	PDX	[19]
	Erlotinib, Gefitinib	EGFR	Cell line	[70]
	BYL719	PI3Kα	Cell line	[71]
	Cisplatin, Carboplatin	Interferes with DNA damage repair mechanism	Cell line	[72]
ESCC	Lapatinib	EGFR, HER2	Cell line	[73]
Gastrointestinal stromal tumour	Imatinib mesylate	v-Abl, c-KIT, PDGFR	Cell line	[74]
Neuroblastoma	TAE684, LDK378	ALK	Cell line	[75]
Rhabdomyosarcoma	MAB391	IGF-IR	Cell line	[76]
Acute myeloid leukaemia	PKC412	PKCα/β/γ, Syk, Flk-1, AKT, PKA, c-Kit, c-Fgr, c-Src, FLT3, PDFRβ, and VEGFR1/2	Cell line	[77]
	AC220	FLT3	Cell line	[77]
	Doxorubicin plus cytosine arabinoside	Inhibition of DNA topoisomerase II activity and synthesis of DNA	Human, Cell line	[53]
	Cisplatin	Interferes with DNA damage repair mechanism	Cell line	[53]

Abbreviations: ALK, Anaplastic lymphoma kinase; c-Met, hepatocyte growth factor receptor; EGFR, epidermal growth factor receptor; ESCC, oesophageal squamous-cell carcinomas; FLT3, fms-like tyrosine kinase 3; HER2, human epidermal growth factor receptor 2; HER3, human epidermal growth factor receptor 3; IGF-1R, insulin-like growth factor 1 receptor; NSCLC, non-small cell lung cancer; PDGFR, platelet-derived growth factor receptor; PDX, patient-derived xenograft models; PI3Kα, phosphoinositide 3-kinase alpha; VEGFR, vascular endothelial growth factor receptor.

**Table 2:** Clinical status of Axl selective drugs.

Drug	Clinical Trial No	Phase	Cancer Type	Monotherapy/Combination
Bemcentinib (BGB324, R428)	NCT03184558	II	TNBC	+Pembrolizumab
	NCT02424617	I/II	NSCLC	+Erlotinib
	NCT03824080	II	AML, MDS	Monotherapy
	NCT03649321	Ib/II	Pancreatic cancer	±Nab-paclitaxel/gemcitabine/cisplatin
	NCT02488408	Ib/II	AML, MDS	±Cytarabine/decitabine
	NCT02872259	Ib/II	Metastatic melanoma	+Pembrolizumab; +Dabrafenib and trametinib
	NCT03184571	II	NSCLC	+Pembrolizumab
	NCT03965494	I	Glioblastoma	Monotherapy, before and after surgery
Dubermatinib (TP-0903)	NCT03572634	I/II	CLL	±Ibrutinib
	NCT02729298	I	Advanced solid tumours (Advanced solid tumours, EGFR positive NSCLC, Colorectal carcinoma, Recurrent ovarian carcinoma, BRAF-mutated melanoma	Monotherapy
DS-1205	NCT03255083	I	Metastatic or unresectable EGFR-mutant NSCLC	+Osimertinib
	NCT03599518	I	Metastatic or unresectable EGFR-mutant NSCLC	+Gefitinib
BA3011 (CAB-AXL-ADC)	NCT03425279	I/II	Solid tumours (NSCLC, Pancreatic cancer, Melanoma, Ewing sarcoma, Osteosarcoma, Leiomyosarcoma, Synovial sarcoma, Liposarcoma, Soft tissue sarcoma, Bone sarcoma, Refractory sarcoma)	±Nivolumab
	NCT04681131	II	NSCLC	±PD-1 inhibitor
Enapotamab vedotin (HuMax-AXL-ADC)	NCT02988817	I/II	Ovarian, Cervical, Endometrial, NSCLC, Thyroid, Melanoma, Sarcoma	Monotherapy
CCT301-38	NCT03393936	I/II	Recurrent or refractory stage IV renal cell carcinoma	Monotherapy
SLC-391	NCT04004442	I	Solid tumours	Monotherapy
AVB-S6-500	NCT04004442	I/II	Advanced urothelial carcinoma	Monotherapy
	NCT03639246	Ib/II	Platinum-resistant recurrent ovarian cancer	+Pegylated liposomal-doxorubicin or paclitaxel
	NCT04019288	I/II	Platinum-resistant or recurrent Ovarian, Fallopian tube, or Primary peritoneal cancer	+Durvalumab (MEDI4736)
	NCT03607955	Ib	Stage III or IV Epithelial ovarian, Primary peritoneal, or Fallopian tube cancer receiving neoadjuvant chemotherapy	+Paclitaxel and carboplatin

Information was obtained from www.clinicaltrials.gov, accessed on 5 January 2021. Abbreviations: AML, acute myeloid leukaemia; CLL, chronic lymphocytic leukaemia; EGFR, epidermal growth factor receptor; MDS, Myelodysplastic syndromes; NSCLC, non-small cell lung cancer; TNBC, triple-negative breast cancer.

**Table 3:** Clinical status of multitargeted drugs inhibiting Axl.

Drug	Target (s)	Clinical Trial No	Phase	Cancer Type	Monotherapy/Combination
Sitravatinib (MGCD516)	VEGFR, PDGFR c-KIT, DDR2, EPHA, FLT3, MET, TYRO3, Axl, and MER	NCT03680521	II	Clear cell RCC	+Nivolumab
		NCT03906071	III	Metastatic NSCLC	+Nivolumab, Docetaxel
		NCT04123704	II	Metastatic TBNC	Monotherapy
		NCT02978859	II	Advanced liposarcoma and other soft tissue sarcomas	Monotherapy
		NCT03606174	II	Urothelial carcinoma	+Nivolumab
		NCT03941873	I/II	Hepatocellular carcinoma, Gastric/Gastroesophageal junction cancer	±Tislelizumab
		NCT03575598	I	HNSCC, Squamous cell carcinoma mouth, Squamous cell carcinoma of the oral cavity	+Nivolumab
		NCT02219711	I/Ib	Advanced solid tumours	Monotherapy
BMS-777607 (ASLAN002)	c-Met, Axl, RON, TYRO3	NCT00605618	I/II	Advanced solid tumours	Monotherapy
		NCT01721148	I	Advanced metastatic tumours	Monotherapy
RXDX-106 (CEP-40783)	Axl, TYRO3, MER, c-Met	NCT03454243	I	Advanced or metastatic solid tumours	Monotherapy
LY2801653 (Merestinib)	c-Met, Axl, RON, MER	NCT02711553	II	Advanced or metastatic biliary tract cancer	Ramucirumab or merestinib or placebo, +cisplatin and gemcitabine
		NCT02920996	II	NSCLC	Monotherapy
		NCT03027284	I	Advanced or metastatic cancer	±other anti-cancer agents
Q702	Axl, MER, CSF1R	NCT04648254	I	Advanced solid tumour	Monotherapy
ONO-7475	Axl, MER	NCT03176277	I/II	Elapsed or refractory AML	±Venetoclax
INCB081776	Axl, MER	NCT03522142	I	Advanced solid tumour	±INCMGA00012

Information was obtained from www.clinicaltrials.gov, accessed on 5 January 2021. Abbreviations: AML, acute myeloid leukaemia; c-Met, hepatocyte growth factor receptor; DDR2, discoidin domain receptor tyrosine kinase 2; EPHA, Ephrin type-A receptor; FLT3, fms-like tyrosine kinase 3; MET, hepatocyte growth factor receptor; NSCLC, non-small cell lung cancer; PDGFR, platelet-derived growth factor receptor; RCC, renal cell carcinoma; RON, macrophage-stimulating protein receptor; TBNC, triple-negative breast cancer; VEGFR, vascular endothelial growth factor receptor.

## References

[1] Gottesman M.M. (2002). Mechanisms of Cancer Drug Resistance. Annu. Rev. Med..

[2] Mansoori B., Mohammadi A., Davudian S., Shirjang S., Baradaran B. (2017). The Different Mechanisms of Cancer Drug Resistance: A Brief Review. Adv. Pharm. Bull..

[3] Housman G., Byler S., Heerboth S., Lapinska K., Longacre M., Snyder N., Sarkar S. (2014). Drug resistance in cancer: An overview. Cancers.

[4] Paul M.K., Mukhopadhyay A.K. (2004). Tyrosine kinase—Role and significance in Cancer. Int. J. Med. Sci..

[5] Corcoran C., O’Driscoll L. (2015). Receptor Tyrosine Kinases and Drug Resistance: Development and Characterization of In Vitro Models of Resistance to RTK inhibitors. Methods Mol. Biol..

[6] Jiao Q., Bi L., Ren Y., Song S., Wang Q., Wang Y. (2018). shan Advances in studies of tyrosine kinase inhibitors and their acquired resistance. Mol. Cancer.

[7] Gjerdrum C., Tiron C., Høiby T., Stefansson I., Haugen H., Sandal T., Collet K., Li S., McCormack E., Gjertsen B.T. (2010). Axl is an essential epithelial-to-mesenchymal transition-induced regulator of breast cancer metastasis and patient survival. Proc. Natl. Acad. Sci. USA.

[8] Dransfield I., Farnworth S. (2016). Axl and Mer Receptor Tyrosine Kinases: Distinct and Nonoverlapping Roles in Inflammation and Cancer. Apoptosis in Cancer Pathogenesis and Anti-Cancer Therapy.

[9] Colavito S.A. (2020). AXL as a Target in Breast Cancer Therapy. J. Oncol..

[10] Zhu C., Wei Y., Wei X. (2019). AXL receptor tyrosine kinase as a promising anti-cancer approach: Functions, molecular mechanisms and clinical applications. Mol. Cancer.

[11] Okimoto R.A., Bivona T.G. (2015). AXL receptor tyrosine kinase as a therapeutic target in NSCLC. Lung Cancer Targets Ther..

[12] Rankin E.B., Fuh K.C., Taylor T.E., Krieg A.J., Musser M., Yuan J., Wei K., Kuo C.J., Longacre T.A., Amato G.J. (2010). AXL is an Essential Factor and Therapeutic Target for Metastatic Ovarian Cancer. Cancer Res..

[13] Korshunov V.A. (2013). Axl-dependent sigaling: A clinical update. Clin. Sci..

[14] Caberoy N.B., Zhou Y., Li W. (2010). Tubby and tubby-like protein 1 are new MerTK ligands for phagocytosis. EMBO J..

[15] Axelrod H., Pienta K.J. (2014). Axl as a mediator of cellular growth and survival. Oncotarget.

[16] Xu M.Z., Chan S.W., Liu A.M., Wong K.F., Fan S.T., Chen J., Poon R.T., Zender L., Lowe S.W., Hong W. (2011). AXL receptor kinase is a mediator of YAP-dependent oncogenic functions in hepatocellular carcinoma. Oncogene.

[17] Mudduluru G., Vajkoczy P., Allgayer H. (2010). Myeloid zinc finger 1 induces migration, invasion, and in vivo metastasis through Axl gene expression in solid cancer. Mol. Cancer Res..

[18] Bdarny M., Prasad M., Balaban N., Ben-Zion J., Dinur A.B., Grénman R., Cohen L., Elkabets M. (2018). The AP-1 complex regulates AXL expression and determines sensitivity to PI3Kα inhibition in esophagus and head and neck squamous cell carcinoma. bioRxiv.

[19] Brand T.M., Lida M., Stein A.P., Corrgan K.L., Braverman C.M., Luthar N., Toulany M., Gill P.S., Salgia R., Kimple R.J. (2014). AXL mediates resistance to cetuximab therapy. Cancer Res..

[20] Vaughan C.A., Singh S., Windle B., Yeudall W.A., Frum R., Grossman S.R., Deb S.P., Deb S. (2012). Gain-of-Function Activity of Mutant p53 in Lung Cancer through Up-Regulation of Receptor Protein Tyrosine Kinase Axl. Genes Cancer.

[21] Hua W., Zhao Y., Jin X., Yu D., He J., Xie D., Duan P. (2018). METTL3 promotes ovarian carcinoma growth and invasion through the regulation of AXL translation and epithelial to mesenchymal transition. Gynecol. Oncol..

[22] Brown M., Black J.R.M., Sharma R., Stebbing J., Pinato D.J. (2016). Gene of the month: Axl. J. Clin. Pathol..

[23] Lin S.H., Wang J., Saintigny P., Wu C.-C., Giri U., Zhang J., Menju T., Diao L., Byers L., Weinstein J.N. (2014). Genes suppressed by DNA methylation in non-small cell lung cancer reveal the epigenetics of epithelial–mesenchymal transition. BMC Genom..

[24] Paccez J.D., Duncan K., Sekar D., Correa R.G., Wang Y., Gu X., Bashin M., Chibale K., Libermann T.A., Zerbini L.F. (2019). Dihydroartemisinin inhibits prostate cancer via JARID2/miR-7/miR-34a-dependent downregulation of Axl. Oncogenesis.

[25] Jiang N., Wang X., Xie X., Liao Y., Liu N., Liu J., Miao N., Shen J., Peng T. (2017). lncRNA DANCR promotes tumor progression and cancer stemness features in osteosarcoma by upregulating AXL via miR-33a-5p inhibition. Cancer Lett..

[26] Kawasaki Y., Miyamoto M., Oda T., Matsumura K., Negishi L., Nakato R., Suda S., Yokota N., Shirahige K., Akiyama T. (2019). The novel lncRNA CALIC upregulates AXL to promote colon cancer metastasis. EMBO Rep..

[27] Valverde P. (2005). Effects of Gas6 and hydrogen peroxide in Axl ubiquitination and downregulation. Biochem. Biophys. Res. Commun..

[28] Hafizi S., Dahlback B. (2006). Signalling and functional diversity within the Axl subfamily of receptor tyrosine kinases. Cytokine Growth Factor Rev..

[29] Tai K.-Y., Shieh Y.-S., Lee C.-S., Shiah S.-G., Wu C.-W. (2008). Axl promotes cell invasion by inducing MMP-9 activity through activation of NF-κB and Brg-1. Oncogene.

[30] Bellosta P., Costa M., Lin D.A., Basilico C. (1995). The receptor tyrosine kinase ARK mediates cell aggregation by homophilic binding. Mol. Cell. Biol..

[31] Meyer A.S., Miller M.A., Gertler F.B., Lauffenburger D.A. (2013). The receptor AXL diversifies EGFR signaling and limits the response to EGFR-targeted inhibitors in triple-negative breast cancer cells. Sci. Signal..

[32] Vouri M., Croucher D.R., Kennedy S.P., An Q., Pilkington G.J., Hafizi S. (2016). Axl-EGFR receptor tyrosine kinase hetero-interaction provides EGFR with access to pro-invasive signalling in cancer cells. Oncogenesis.

[33] Goyette M.A., Duhamel S., Aubert L., Pelletier A., Savage P., Thibault M.P., Johnson R.M., Carmeliet P., Basik M., Gaboury L. (2018). The Receptor Tyrosine Kinase AXL Is Required at Multiple Steps of the Metastatic Cascade during HER2-Positive Breast Cancer Progression. Cell Rep..

[34] Huang J.S., Cho C.Y., Hong C.C., De Yan M., Hsieh M.C., Lay J.D., Lai G.M., Cheng A.L., Chuang S.E. (2013). Oxidative Stress Enhances Axl-Mediated Cell Migration through an Akt1/Rac1-Dependent Mechanism.

[35] Oien D.B., Garay T., Eckstein S., Chien J. (2018). Cisplatin and pemetrexed activate AXL and AXL inhibitor BGB324 enhances mesothelioma cell death from chemotherapy. Front. Pharmacol..

[36] Hanahan D., Weinberg R.A. (2011). Hallmarks of cancer: The next generation. Cell.

[37] Lee C.-H., Yen C.-Y., Liu S.-Y., Chen C.-K., Chiang C.-F., Shiah S.-G., Chen P.-H., Shieh Y.-S. (2012). Axl Is a Prognostic Marker in Oral Squamous Cell Carcinoma. Ann. Surg. Oncol..

[38] Corno C., Gatti L., Lanzi C., Zaffaroni N., Colombo D., Perego P. (2016). Role of the Receptor Tyrosine Kinase Axl and its Targeting in Cancer Cells. Curr. Med. Chem..

[39] Paccez J.D., Vogelsang M., Parker M.I., Zerbini L.F. (2014). The receptor tyrosine kinase Axl in cancer: Biological functions and therapeutic implications. Int. J. Cancer.

[40] Paccez J.D., Vasques G.J., Correa R.G., Vasconcellos J.F., Duncan K., Gu X., Bhasin M., Libermann T.A., Zerbini L.F. (2013). The receptor tyrosine kinase Axl is an essential regulator of prostate cancer proliferation and tumor growth and represents a new therapeutic target. Oncogene.

[41] Sinha S., Boysen J., Nelson M., Secreto C., Warner S.L., Bearss D.J., Lesnick C., Shanafelt T.D., Kay N.E., Ghosh A.K. (2015). Targeted Axl inhibition primes chronic lymphocytic leukemia B cells to apoptosis and shows synergistic/additive effects in combination with BTK inhibitors. Clin. Cancer Res..

[42] Zhu S., Liu G., Fu W., Hu J., Fu K., Jia W. (2017). Axl promotes the proliferation, invasion and migration of Wilms’ tumor and can be used as a prognostic factor. OncoTargets. Ther..

[43] Martinho O., Zucca L.E., Reis R.M. (2015). AXL as a modulator of sunitinib response in glioblastoma cell lines. Exp. Cell Res..

[44] Zajac O., Leclere R., Nicolas A., Meseure D., Marchiò C., Vincent-Salomon A., Roman-Roman S., Schoumacher M., Dubois T. (2020). AXL Controls Directed Migration of Mesenchymal Triple-Negative Breast Cancer Cells. Cells.

[45] Zhang X., Yang L., Szeto P., Zhang Y., Amarasinghe K., Li J., McLean C., Shackleton M., Harvey K.F. (2020). The Hippo pathway oncoprotein YAP promotes melanoma cell invasion and spontaneous metastasis. Oncogene.

[46] Asiedu M.K., Beauchamp-Perez F.D., Ingle J.N., Behrens M.D., Radisky D.C., Knutson K.L. (2014). AXL induces epithelial-to-mesenchymal transition and regulates the function of breast cancer stem cells. Oncogene.

[47] Wang X., Beitler J.J., Huang W., Chen G., Qian G., Magliocca K., Patel M., Chen A.Y., Zhang J., Nannapaneni S. (2018). Honokiol radiosensitizes squamous cell carcinoma of the head and neck by downregulation of survivin. Clin. Cancer Res..

[48] Koorstra J.-B.M., Karikari C.A., Feldmann G., Bisht S., Rojas P.L., Offerhaus G.J.A., Alvarez H., Maitra A. (2009). The Axl receptor tyrosine kinase confers an adverse prognostic influence in pancreatic cancer and represents a new therapeutic target. Cancer Biol. Ther..

[49] Lee Y., Ko D., Min H.-J., Kim S.B., Ahn H.-M., Lee Y., Kim S. (2016). TMPRSS4 induces invasion and proliferation of prostate cancer cells through induction of Slug and cyclin D1. Oncotarget.

[50] Li Y., Ye X., Tan C., Hongo J.A., Zha J., Liu J., Kallop D., Ludlam M.J.C., Pei L. (2009). Axl as a potential therapeutic target in cancer: Role of Axl in tumor growth, metastasis and angiogenesis. Oncogene.

[51] Rankin E.B., Giaccia A.J. (2016). The receptor tyrosine kinase AXL in cancer progression. Cancers.

[52] Jin Y., Nie D., Li J., Du X., Lu Y., Li Y., Liu C., Zhou J., Pan J. (2017). Gas6/AXL signaling regulates self-renewal of chronic myelogenous leukemia stem cells by stabilizing β-catenin. Clin. Cancer Res..

[53] Hong C.-C.C., Lay J.-D.D., Huang J.-S.S., Cheng A.-L.L., Tang J.-L.L., Lin M.-T.T., Lai G.-M.M., Chuang S.-E.E. (2008). Receptor tyrosine kinase AXL is induced by chemotherapy drugs and overexpression of AXL confers drug resistance in acute myeloid leukemia. Cancer Lett..

[54] Aguilera T.A., Giaccia A.J. (2017). Molecular pathways: Oncologic pathways and their role in T-cell exclusion and immune evasion—A new role for the AXL receptor tyrosine kinase. Clin. Cancer Res..

[55] Tsukita Y., Fujino N., Miyauchi E., Saito R., Fujishima F., Itakura K., Kyogoku Y., Okutomo K., Yamada M., Okazaki T. (2019). Axl kinase drives immune checkpoint and chemokine signalling pathways in lung adenocarcinomas. Mol. Cancer.

[56] Aguilera T.A., Rafat M., Castellini L., Shehade H., Kariolis M.S., Hui A.B.Y., Stehr H., Von Eyben R., Jiang D., Ellies L.G. (2016). Reprogramming the immunological microenvironment through radiation and targeting Axl. Nat. Commun..

[57] Zhang G., Wang M., Zhao H., Cui W. (2018). Function of Axl receptor tyrosine kinase in non-small cell lung cancer. Oncol. Lett..

[58] Taniguchi H., Yamada T., Wang R., Tanimura K., Adachi Y., Nishiyama A., Tanimoto A., Takeuchi S., Araujo L.H., Boroni M. (2019). AXL confers intrinsic resistance to osimertinib and advances the emergence of tolerant cells. Nat. Commun..

[59] Kim H.R., Kim W.S., Choi Y.J., Choi C.M., Rho J.K., Lee J.C. (2013). Epithelial-mesenchymal transition leads to crizotinib resistance in H2228 lung cancer cells with EML4-ALK translocation. Mol. Oncol..

[60] Linger R.M.A., Cohen R.A., Cummings C.T., Sather S., Migdall-Wilson J., Middleton D.H.G., Lu X., Barón A.E., Franklin W.A., Merrick D.T. (2013). Mer or Axl receptor tyrosine kinase inhibition promotes apoptosis, blocks growth and enhances chemosensitivity of human non-small cell lung cancer. Oncogene.

[61] Wu F., Li J., Jang C., Wang J., Xiong J. (2014). The role of Axl in drug resistance and epithelial-to-mesenchymal transition of non-small cell lung carcinoma. Int. J. Clin. Exp. Pathol..

[62] Zhang Z., Lee J.C., Lin L., Olivas V., Au V., LaFramboise T., Abdel-Rahman M., Wang X., Levine A.D., Rho J.K. (2012). Activation of the AXL kinase causes resistance to EGFR-targeted therapy in lung cancer. Nat. Genet..

[63] Lin J.-Z., Wang Z.-J., De W., Zheng M., Xu W.-Z., Wu H.-F., Armstrong A., Zhu J.-G. (2017). Targeting AXL overcomes resistance to docetaxel therapy in advanced prostate cancer. Oncotarget.

[64] Liu L., Greger J., Shi H., Liu Y., Greshock J., Annan R., Halsey W., Sathe G.M., Martin A.-M.M., Gilmer T.M. (2009). Novel mechanism of lapatinib resistance in HER2-positive breast tumor cells: Activation of AXL. Cancer Res..

[65] Creedon H., Gómez-Cuadrado L., Tarnauskaite Ž., Balla J., Canel M., MacLeod K.G., Serrels B., Fraser C., Unciti-Broceta A., Tracey N. (2016). Identification of novel pathways linking epithelial-to-mesenchymal transition with resistance to HER2-targeted therapy. Oncotarget.

[66] Li Y., Jia L., Liu C., Gong Y., Ren D., Wang N., Zhang X., Zhao Y. (2015). Axl as a downstream effector of TGF-β1 via PI3K/Akt-PAK1 signaling pathway promotes tumor invasion and chemoresistance in breast carcinoma. Tumor Biol..

[67] Zhao Y., Sun X., Jiang L., Yang F., Zhang Z., Jia L. (2012). Differential expression of Axl and correlation with invasion and multidrug resistance in cancer cells. Cancer Investig..

[68] Gusenbauer S., Vlaicu P., Ullrich A. (2013). HGF induces novel EGFR functions involved in resistance formation to tyrosine kinase inhibitors. Oncogene.

[69] Pinato D.J., Brown M.W., Trousil S., Aboagye E.O., Beaumont J., Zhang H., Coley H.M., Mauri F.A., Sharma R. (2019). Integrated analysis of multiple receptor tyrosine kinases identifies Axl as a therapeutic target and mediator of resistance to sorafenib in hepatocellular carcinoma. Br. J. Cancer.

[70] Giles K.M., Kalinowski F.C., Candy P.A., Epis M.R., Zhang P.M., Redfern A.D., Stuart L.M., Goodal G.J., Leedman P.J. (2013). Axl mediates acquired resistance of head and neck cancer cells to the epidermal growth factor receptor inhibitor erlotinib. Mol. Cancer Ther..

[71] Elkabets M., Pazarentzos E., Juric D., Sheng Q., Raphael A., Brook S., Benzaken A.O., Rodon J., Morse N., Yan J. (2015). AXL mediates resistance to PI3Kα inhibition by activating the EGFR/PKC/mTOR axis in head and neck and esophageal squamous cell carcinomas. Cancer Cell.

[72] Brand T.M., Stein A.P., Corrigan K.L., Braverman C.M., Coan J., Pearson H.E., Bahrar H., Fowler T.L., Bednarz B.P., Saha S. (2015). AXL is a logical molecular target in head and neck squamous cell carcinoma. Clin. Cancer Res..

[73] Hsieh M.-S., Yang P.-W., Wong L.-F., Lee J.-M. (2016). The AXL receptor tyrosine kinase is associated with adverse prognosis and distant metastasis in esophageal squamous cell carcinoma. Oncotarget.

[74] Mahadevan D., Cooke L., Riley C., Swart R., Simons B., Croce K.D., Wisner L., Iorio M., Shakalya K., Garewal H. (2007). A novel tyrosine kinase switch is a mechanism of imatinib resistance in gastrointestinal stromal tumors. Oncogene.

[75] Debruyne D.N., Bhatnagar N., Sharma B., Luther W., Moore N.F., Cheung N.-K., Gray N.S., George R.E. (2015). ALK inhibitor resistance in ALK F1174L -driven neuroblastoma is associated with AXL activation and induction of EMT. Oncogene.

[76] Huang F., Hurlburt W., Greer A., Reeves K.A., Hillerman S., Chang H., Fargnoli J., Finckenstein F.G., Gottardis M.M., Carboni J.M. (2010). Differential Mechanisms of Acquired Resistance to Insulin- like Growth Factor-I Receptor Antibody Therapy or to a Small-Molecule Inhibitor, BMS-754807, in a Human Rhabdomyosarcoma Model. Cancer Res. AACR.

[77] Park I.K., Mundy-Bosse B., Whitman S.P., Zhang X., Warner S.L., Bearss D.J., Blum W., Marcucci G., Caligiuri M.A. (2015). Receptor tyrosine kinase Axl is required for resistance of leukemic cells to FLT3-targeted therapy in acute myeloid leukemia. Leukemia.

[78] Jiao Y., Ou W., Meng F., Zhou H., Wang A. (2011). Targeting HSP90 in ovarian cancers with multiple receptor tyrosine kinase coactivation. Mol. Cancer.

[79] Ferlay J., Colombet M., Soerjomataram I., Mathers C., Parkin D.M.M., Piñeros M., Znaor A., Bray F. (2019). Estimating the global cancer incidence and mortality in 2018: GLOBOCAN sources and methods. Int. J. Cancer.

[80] Herbst R.S., Heymach J.V., Lippman S.M. (2008). Lung cancer. N. Engl. J. Med..

[81] Howlader N., Noone A.M., Krapcho M., Miller D., Brest A., Yu M., Ruhl J., Tatalovich Z., Mariotto A., Lewis D.R. (2020). SEER Cancer Statistics Review, 1975–2017.

[82] Jänne P.A., Yang J.C.-H., Kim D.-W., Planchard D., Ohe Y., Ramalingam S.S., Ahn M.-J., Kim S.-W., Su W.-C., Horn L. (2015). AZD9291 in EGFR inhibitor-resistant non-small-cell lung cancer. N. Engl. J. Med..

[83] Dungo R.T., Keating G.M. (2013). Afatinib: First global approval. Drugs.

[84] Lynch T.J., Bell D.W., Sordella R., Gurubhagavatula S., Okimoto R.A., Brannigan B.W., Harris P.L., Haserlat S.M., Supko J.G., Haluska F.G. (2004). Activating mutations in the epidermal growth factor receptor underlying responsiveness of non-small-cell lung cancer to gefitinib. N. Engl. J. Med..

[85] Paez J.G., Jänne P.A., Lee J.C., Tracy S., Greulich H., Gabriel S., Herman P., Kaye F.J., Lindeman N., Boggon T.J. (2004). EGFR mutations in lung cancer: Correlation with clinical response to gefitinib therapy. Science.

[86] Byers L.A., Diao L., Wang J., Saintigny P., Girard L., Peyton M., Shen L., Fan Y., Giri U., Tumula P.K. (2013). An epithelial-mesenchymal transition gene signature predicts resistance to EGFR and PI3K inhibitors and identifies Axl as a therapeutic target for overcoming EGFR inhibitor resistance. Clin. Cancer Res..

[87] Wang S., Pang T., Gao M., Kang H., Ding W., Sun X., Zhao Y., Zhu W., Tang X., Yao Y. (2013). HPV E6 induces eIF4E transcription to promote the proliferation and migration of cervical cancer. FEBS Lett..

[88] Ji W., Choi C.-M., Rho J.K., Jang S.J., Park Y.S., Chun S.-M., Kim W.S., Lee J.-S., Kim S.-W., Lee D.H. (2013). Mechanisms of acquired resistance to EGFR-tyrosine kinase inhibitor in Korean patients with lung cancer. BMC Cancer.

[89] Tian Y., Zhang Z., Miao L., Yang Z., Yang J., Wang Y.Y., Qian D., Cai H., Wang Y.Y. (2016). Anexelekto (AXL) Increases Resistance to EGFR-TKI and Activation of AKT and ERK1/2 in Non-Small Cell Lung Cancer Cells. Oncol. Res. Featur. Preclin. Clin. Cancer Ther..

[90] Du W., Sun L., Liu T., Zhu J., Zeng Y., Zhang Y., Wang X., Liu Z., Huang J.A. (2020). The miR_625_3p/AXL axis induces non_T790M acquired resistance to EGFR_TKI via activation of the TGF_β/Smad pathway and EMT in EGFR_mutant non_small cell lung cancer. Oncol. Rep..

[91] Jeong I., Song J., Bae S.Y., Lee S.K. (2019). Overcoming the Intrinsic Gefitinib-resistance via Downregulation of AXL in Non-small Cell Lung Cancer. J. Cancer Prev..

[92] Jackman D., Pao W., Riely G.J., Engelman J.A., Kris M.G., Jänne P.A., Lynch T., Johnson B.E., Miller V.A. (2010). Clinical Definition of Acquired Resistance to Epidermal Growth Factor Receptor Tyrosine Kinase Inhibitors in Non–Small-Cell Lung Cancer. J. Clin. Oncol..

[93] Wu Z., Bai F., Fan L., Pang W., Han R., Wang J., Liu Y., Yan X., Duan H., Xing L. (2015). Coexpression of receptor tyrosine kinase AXL and EGFR in human primary lung adenocarcinomas. Hum. Pathol..

[94] Suda K., Mizuuchi H., Sato K., Takemoto T., Iwasaki T., Mitsudomi T. (2014). The insulin-like growth factor 1 receptor causes acquired resistance to erlotinib in lung cancer cells with the wild-type epidermal growth factor receptor. Int. J. Cancer.

[95] Thienelt C.D., Bunn P.A., Hanna N., Rosenberg A., Needle M.N., Long M.E., Gustafson D.L., Kelly K. (2005). Multicenter phase I/II study of cetuximab with paclitaxel and carboplatin in untreated patients with stage IV non-small-cell lung cancer. J. Clin. Oncol..

[96] Baselga J. (2001). The EGFR as a target for anticancer therapy--focus on cetuximab. Eur. J. Cancer.

[97] Brand T.M., Iida M., Wheeler D.L. (2011). Molecular mechanisms of resistance to the EGFR monoclonal antibody cetuximab. Cancer Biol. Ther..

[98] Terry S., Abdou A., Engelsen A.S.T., Buart S., Dessen P., Corgnac S., Collares D., Meurice G., Gausdal G., Baud V. (2019). AXL Targeting Overcomes Human Lung Cancer Cell Resistance to NK- and CTL-Mediated Cytotoxicity. Cancer Immunol. Res..

[99] Siegel R.L., Miller K.D., Jemal A. (2018). Cancer statistics, 2018. CA Cancer J. Clin..

[100] Denis L., Murphy G.P. (1993). Overview of phase III trials on combined androgen treatment in patients with metastatic prostate cancer. Cancer.

[101] Ringel I., Horwitz S.B. (1991). Studies with RP 56976 (taxotere): A semisynthetic analogue of taxol. J. Natl. Cancer Inst..

[102] Bumbaca B., Li W. (2018). Taxane resistance in castration-resistant prostate cancer: Mechanisms and therapeutic strategies. Acta Pharm. Sin. B.

[103] Bray F., Ferlay J., Soerjomataram I., Siegel R.L., Torre L.A., Jemal A. (2018). Global cancer statistics 2018: GLOBOCAN estimates of incidence and mortality worldwide for 36 cancers in 185 countries. CA. Cancer J. Clin..

[104] Shen Y., Zhang W., Liu J., He J., Cao R., Chen X., Peng X., Xu H., Zhao Q., Zhong J. (2019). Therapeutic activity of DCC-2036, a novel tyrosine kinase inhibitor, against triple-negative breast cancer patient-derived xenografts by targeting AXL/MET. Int. J. Cancer.

[105] Caldas-Lopes E., Cerchietti L., Ahn J.H., Clement C.C., Robles A.I., Rodina A., Moulick K., Taldone T., Gozrnan A., Guo Y. (2009). Hsp90 inhibitor PU-H71, a multimodal inhibitor of malignancy, induces complete responses in triple-negative breast cancer models. Proc. Natl. Acad. Sci. USA.

[106] Miller M.A., Oudin M.J., Sullivan R.J., Wang S.J., Meyer A.S., Im H., Frederick D.T., Tadros J., Griffith L.G., Lee H. (2016). Reduced proteolytic shedding of receptor tyrosine kinases is a post-translational mechanism of kinase inhibitor resistance. Cancer Discov..

[107] Wang C., Jin H., Wang N., Fan S., Wang Y., Zhang Y., Wei L., Tao X., Gu D., Zhao F. (2016). Gas6/Axl axis contributes to chemoresistance and metastasis in breast cancer through Akt/GSK-3β/β- catenin signaling. Theranostics.

[108] Center M.M., Jemal A. (2011). International trends in liver cancer incidence rates. Cancer Epidemiol. Biomark. Prev..

[109] Vogel A., Saborowski A. (2020). Current strategies for the treatment of intermediate and advanced hepatocellular carcinoma. Cancer Treat. Rev..

[110] Reichl P., Dengler M., van Zijl F., Huber H., Führlinger G., Reichel C., Sieghart W., Peck-Radosavljevic M., Grubinger M., Mikulits W. (2015). Axl activates autocrine transforming growth factor-β signaling in hepatocellular carcinoma. Hepatology.

[111] Lee H.J., Jeng Y.M., Chen Y.L., Chung L., Yuan R.H. (2014). Gas6/Axl pathway promotes tumor invasion through the transcriptional activation of slug in hepatocellular carcinoma. Carcinogenesis.

[112] Giannelli G., Villa E., Lahn M. (2014). Transforming growth factor-β as a therapeutic target in hepatocellular carcinoma. Cancer Res..

[113] Li X.Y., Wen J.Y., Jia C.C., Wang T.T., Li X., Dong M., Lin Q., Chen Z.H., Ma X.K., Wei L. (2015). MicroRNA-34a-5p enhances sensitivity to chemotherapy by targeting AXL in hepatocellular carcinoma MHCC-97L cells. Oncol. Lett..

[114] Leung C.O.N., Tong M., Chung K.P.S., Zhou L., Che N., Tang K.H., Ding J., Lau E.Y.T., Ng I.O.L., Ma S. (2020). Overriding Adaptive Resistance to Sorafenib Through Combination Therapy With Src Homology 2 Domain–Containing Phosphatase 2 Blockade in Hepatocellular Carcinoma. Hepatology.

[115] Felip E., Brunsvig P., Vinolas N., Ponce Aix S., Carcereny Costa E., Dómine Gomez M., Trigo Perez J.M., Arriola E., Campelo R.G., Spicer J.F. (2019). A phase II study of bemcentinib (BGB324), a first-in-class highly selective AXL inhibitor, with pembrolizumab in pts with advanced NSCLC: OS for stage I and preliminary stage II efficacy. J. Clin. Oncol..

[116] Yang G., Sau C., Lai W., Cichon J., Li W. (2014). An engineered Axl decoy receptor effectively silences the Gas6/Axl signaling axis. Nat. Chem. Biol..

[117] Rodon Ahnert J., Taylor M.H., O’Reilly E.M., Zhang J., Doebele R.C., Ben Y., Sharp L.L., Boyle W.J., Chang C., Frey G. (2018). A phase 1/2 dose-escalation and expansion study of a conditionally active anti-AXL humanized monoclonal antibody (BA3011) in patients with advanced solid tumors. J. Clin. Oncol..

[118] Ameratunga M., Harvey R.D., Mau-Sørensen M., Thistlethwaite F., Forssmann U., Gupta M., Johannsdottir H., Ramirez-Andersen T., Bohlbro M.L., Losic N. (2019). First-in-human, dose-escalation, phase (ph) I trial to evaluate safety of anti-Axl antibody-drug conjugate (ADC) enapotamab vedotin (EnaV) in solid tumors. J. Clin. Oncol..

[119] Duan Y., Luo L., Qiao C., Li X., Wang J., Liu H., Zhou T., Shen B., Lv M., Feng J. (2019). A novel human anti-AXL monoclonal antibody attenuates tumour cell migration. Scand. J. Immunol..

[120] Hwang J.A., Hur J.Y., Kim Y., Im J.H., Jin S.H., Ryu S.H., Choi C. (2021). Efficacy of newly discovered DNA aptamers targeting AXL in a lung cancer cell with acquired resistance to Erlotinib. Transl. Cancer Res..

[121] Cerchia L., Esposito C.L., Camorani S., Rienzo A., Stasio L., Insabato L., Affuso A., De Franciscis V. (2012). Targeting Axl with an high-affinity inhibitory aptamer. Mol. Ther..

